# Cultural syndromes in the era of globalization

**DOI:** 10.1192/j.eurpsy.2022.1627

**Published:** 2022-09-01

**Authors:** M.D.C. Molina Liétor, I. Cuevas Iñiguez

**Affiliations:** 1 Hospital Universitario Príncipe de Asturias, Psiquiatría, Alcalá de Henares, Spain; 2 Hospital Principe de Asturias, Psiquiatría, Alcala de Henares, Spain

**Keywords:** migration, cultural syndromes, globalization

## Abstract

**Introduction:**

Cultural syndromes are pathologies that cannot be understood outside the cultural or subcultural context of the person who suffers from it, since both their etiology and symptoms are symbolized by the patient and by the environment in fields of significance inherent to their culture. The globalization process in which we are involved affects the presentation, understanding, diagnosis and treatment of cultural syndromes as they were traditionally understood.

**Objectives:**

The objective of this work is to review the current state of cultural syndromes, the evolution of incidence and prevalence in recent years, as well as whether the globalization process has affected their understanding.

**Methods:**

A bibliographic review has been carried out on cultural syndromes and case reports in both endemic and foreign populations. Likewise, a reflection is made on the possible evolution of these syndromes.

**Results:**

Globalization has been understood as a natural process of integration of nations and their cultures, incorporating the diversity and specificity of the other without forgetting their own and traditional characteristics. Within the globalization process, positive advances in the health area are recognized, specifically in the fields of communication and biotechnology. However, the negative impact of globalization on the daily life and health of people worldwide is undeniable. Those that are economically most disadvantaged are particularly affected. The cases of cultural syndromes in distant countries, the misunderstanding of the symptoms as well as the difficulties of integration of migrant patients with mental suffering must be the object of debate and study.

**Conclusions:**

Globalization affects the care and understanding of mental health

**Disclosure:**

No significant relationships.

